# Exosome-based modulation of ferroptosis in neurological disorders: mechanisms, therapeutic potential, and translational challenges

**DOI:** 10.3389/fimmu.2025.1677808

**Published:** 2025-10-20

**Authors:** Xiaoying Bao, Liwei Chen, Hong Yu, Yunan Xie, Liangxiao Luo, Li Luo, Hanbing Wang, Rongbing Chen, Yongwei Cheng, Da Sun, Chunwu Zhang

**Affiliations:** ^1^ Geriatrics Center, The First Affiliated Hospital of Wenzhou Medical University, Wenzhou, China; ^2^ Cixi Biomedical Research Institute, Wenzhou Medical University, Cixi, China; ^3^ Department of Critical Care Medicine, Yiwu Central Hospital, the Affiliated Yiwu Hospital of Wenzhou Medical University, Yiwu, China; ^4^ Department of Biotechnology, The University of Hong Kong, Kowloon, Hong Kong SAR, China; ^5^ Department of Biomedical Engineering, City University of Hong Kong, Kowloon, Hong Kong SAR, China; ^6^ National Engineering Research Center of Cell Growth Factor Drugs and Protein Biologics, Wenzhou Medical University, Wenzhou, China; ^7^ Institute of Life Sciences & Biomedical Collaborative Innovation Center of Zhejiang Province, Wenzhou University, Wenzhou, China

**Keywords:** ferroptosis, exosomes, neurodegeneration, blood–brain barrier, oxidative stress

## Abstract

Neurological disorders, including acute insults such as stroke and traumatic brain injury and chronic neurodegenerative diseases like Alzheimer’s disease and Parkinson’s disease, exert a profound global health burden. Ferroptosis, a distinct form of regulated cell death driven by iron accumulation, lipid peroxidation, and oxidative stress, has emerged as a central pathological mechanism across these conditions. Exosomes, nanoscale extracellular vesicles capable of crossing the blood-brain barrier and delivering functional cargos such as microRNAs, long non-coding RNAs, and proteins, have demonstrated remarkable potential in modulating ferroptotic signaling. Through regulation of the GPX4–GSH axis, ferritinophagy, iron homeostasis, and antioxidant pathways, exosome-based interventions offer neuroprotective benefits in diverse models of neurological injury. This review synthesizes current advances in the mechanistic understanding of ferroptosis and highlights emerging strategies leveraging exosomes as precision delivery platforms for ferroptosis-targeted therapy. We also discuss the translational challenges and future directions necessary to realize exosome-guided neuroprotection as a viable clinical paradigm.

## 1 Introduction

Neurological disorders, encompassing both acute brain injuries such as ischemic stroke and traumatic brain injury (TBI), and chronic neurodegenerative diseases such as Alzheimer’s disease (AD) and Parkinson’s disease (PD), represent a mounting global health crisis (1). These conditions collectively account for a substantial proportion of mortality and long-term disability worldwide, global DALY counts attributed to these conditions increased by 18.2% (8.7–26.7) between 1990 and 2021, as reported in the Global Burden of Disease Study 2021 (2). Despite their clinical heterogeneity, they share convergent pathophysiological mechanisms, most notably, oxidative stress, neuroinflammation, and iron dysregulation, which synergistically drive progressive neural cell damage and functional decline (3).

The scale of the global burden is striking. Data from the World Stroke Organization and GBD 2021 indicate that stroke affects 93.8 million people globally, with approximately 11.9 million new cases and 7.3 million deaths (10.7% of all deaths) reported in 2021 alone (4). The World Health Organization estimates that TBI, often resulting from falls, vehicular accidents, or sports-related trauma, accounts for an estimated 50–60 million new cases each year and incurs a global economic burden of over $400 billion (5). Meanwhile, Alzheimer’s Disease International (ADI) reports that neurodegenerative diseases such as AD and PD afflict over 50 million people globally, a number projected to surpass 139 million by 2050 due to population aging (6). With the aging of the world’s population, age-related neurodegenerative diseases have become one of the biggest problems to be solved urgently in modern society (7). These conditions are not only devastating to individuals and families but also place extraordinary strain on healthcare systems.

Emerging evidence implicates ferroptosis as a common and critical form of regulated cell death underlying diverse neurological disorders. Ferroptosis is an iron-dependent, non-apoptotic cell death modality characterized by overwhelming lipid peroxidation and reactive oxygen species (ROS) accumulation. Central to this process is the dysregulation of intracellular iron metabolism and the depletion of key antioxidant systems, particularly glutathione (GSH) and its associated enzyme GSH peroxidase 4 (GPX4) (8). In acute neurological insults such as ischemic stroke and TBI, ferroptosis is initiated by iron overload and ROS generation through Fenton chemistry, leading to neuronal injury and secondary damage (9, 10). Ferroptosis plays a pivotal role in the pathogenesis of neurodegenerative diseases, and targeting key regulatory genes involved in this process can effectively delay neurodegeneration (11, 12).

**Figure 1:**
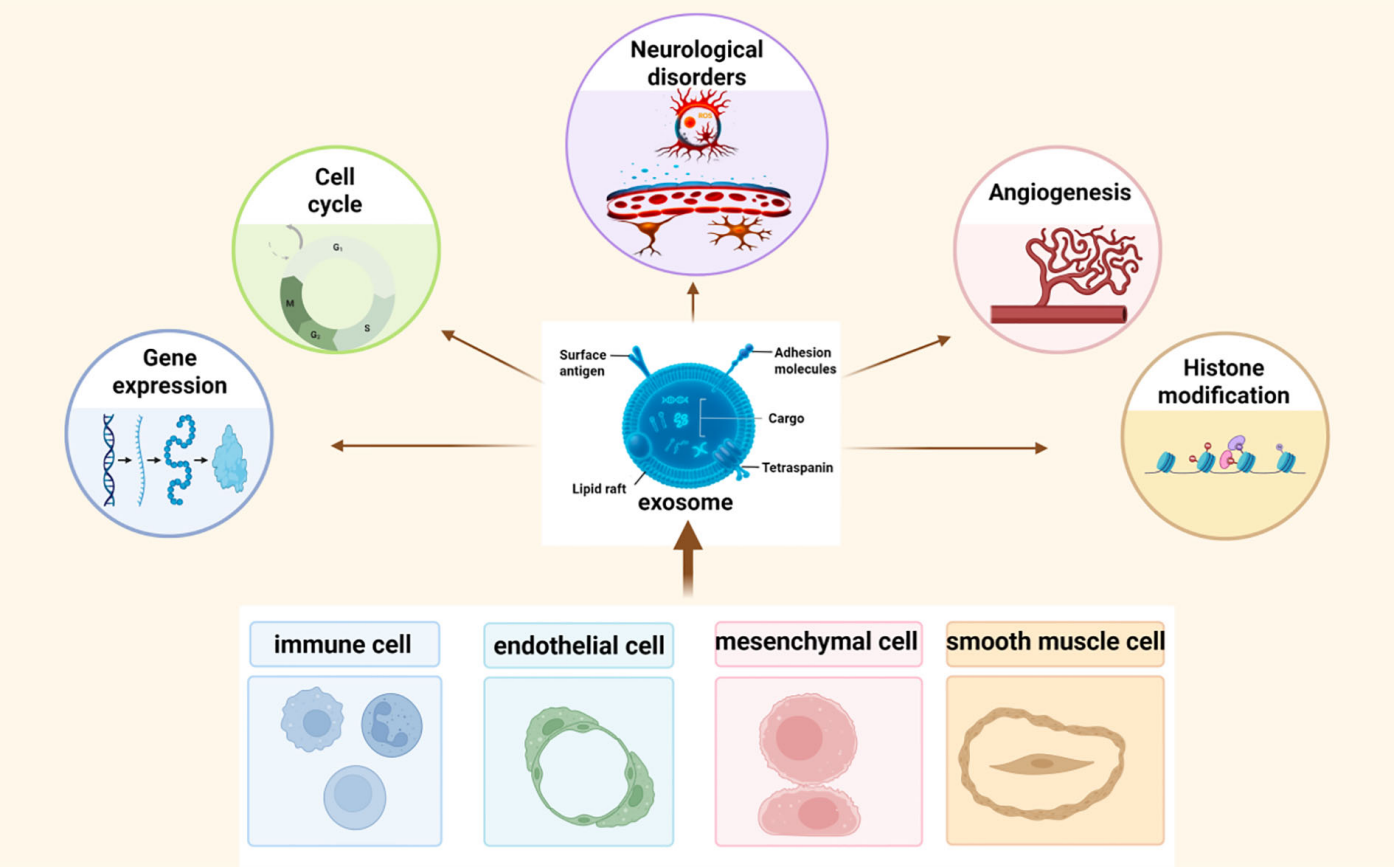
Exosomes are nanoscale extracellular vesicles released by multiple cell types, including endothelial cells, mesenchymal stem cells, immune cells, and smooth muscle cells. They carry bioactive cargos such as proteins, lipids, and non-coding RNAs, and mediate intercellular communication by regulating gene expression, cell survival, angiogenesis, and epigenetic processes within recipient cells.

Given its pivotal role in neuronal vulnerability, ferroptosis has emerged as an attractive target for therapeutic intervention across a spectrum of neurological conditions. Among the innovative strategies under investigation, exosomes, a subtype of extracellular vesicles (40–160 nm) secreted by various cell types, have garnered increasing attention as both biomarkers and delivery vehicles for therapeutic agents (13, 14). Exosomes carry a cargo of bioactive molecules, including proteins, lipids, and nucleic acids (eg., mRNAs, microRNAs (miRNAs), long non-coding RNAs, DNA, etc.), which they transfer between cells to regulate intercellular communication and modulate recipient cell function (13, 15). Their ability to cross physiological barriers such as the blood-brain barrier (BBB), evade immune detection, and deliver functional cargo to specific cell types renders them highly promising candidates for neurotherapeutics (16, 17). Importantly, emerging research suggests that exosomes may exert direct neuroprotective effects by modulating ferroptosis-related pathways (18). Through targeted delivery of antioxidant molecules, iron regulators, and gene-modifying RNAs, exosomes can suppress oxidative stress, restore redox balance, and inhibit ferroptosis-driven neural damage. Compared to synthetic nanocarriers, exosomes offer superior biocompatibility, reduced toxicity, and intrinsic targeting potential, making them a versatile platform for developing next-generation therapies for neurological disorders (**Figure 1**).

Nevertheless, despite the substantial burden of neurological disorders and growing evidence for ferroptosis as a convergent pathological mechanism, the therapeutic potential of exosome-based ferroptosis modulation remains underexplored. Few studies provide systematic comparisons across exosome sources or disease contexts, and clinical validation is still lacking. Addressing these knowledge gaps provides the rationale for this review.

This review aims to systematically elucidate the mechanistic interplay between exosome biology and ferroptosis regulation in neurological diseases. We first dissect the molecular underpinnings of ferroptosis and its contribution to acute and chronic neurodegeneration. We then explore how exosome-mediated delivery of therapeutic cargos—particularly regulatory RNAs and antioxidant proteins—modulates ferroptotic signaling cascades. Finally, we discuss the challenges and innovations in exosome engineering for Central nervous system(CNS)-targeted therapy, offering perspectives on the clinical translation of exosome-based interventions for ferroptosis-driven neurological injury.

## 2 The molecular mechanisms of ferroptosis and its pathological role in neurological disorders

### 2.1 Molecular mechanisms underlying ferroptosis

Ferroptosis is a distinct, iron-dependent form of regulated cell death characterized by the accumulation of lethal lipid peroxides and uncontrolled oxidative stress (19). Unlike apoptosis, necrosis, and autophagy, ferroptosis is fundamentally driven by iron-mediated redox imbalance and extensive lipid membrane damage, and it has been recognized as a central mechanism underlying the progression of various neurological disorders (8, 20, 21). At the molecular level, ferroptosis is primarily orchestrated by three interconnected pathways: iron metabolism, lipid peroxidation, and the cellular antioxidant defense system.

#### 2.1.1 Dysregulation of iron homeostasis

Iron plays a critical role in numerous physiological processes, including DNA synthesis, oxygen transport, and mitochondrial energy metabolism. Physiologically, ferric iron (Fe³^+^) circulates in a redox-inactive form bound to transferrin, whereas ferrous iron (Fe²^+^) is highly reactive and soluble (22). When cellular iron regulation is impaired, excess Fe²^+^ catalyzes the Fenton reaction, producing hydroxyl radicals (·OH) that inflict widespread oxidative damage (23).

Key regulators of systemic and cellular iron homeostasis include hepcidin, which inhibits intestinal iron absorption by promoting the degradation of the iron exporter ferroportin (FPN), and hypoxia-inducible factors (HIFs), which suppress hepcidin under hypoxic conditions, thereby enhancing iron mobilization (24, 25). Overload of labile iron leads to the generation of toxic non-transferrin-bound iron (NTBI), promoting ROS formation and initiating lipid peroxidation cascades (26, 27). Intracellularly, iron is safely sequestered within ferritin complexes. Under pathological conditions, ferritin is degraded via autophagic pathways mediated by nuclear receptor coactivator 4 (NCOA4), a process termed ferritinophagy, liberating iron into the labile pool and exacerbating oxidative stress (22).

#### 2.1.2 Induction of lipid peroxidation

The hallmark of ferroptosis is the iron-catalyzed peroxidation of polyunsaturated fatty acids (PUFAs) incorporated into phospholipids of cellular membranes. Critical enzymes such as acyl-CoA synthetase long-chain family member 4 (ACSL4) and lysophosphatidylcholine acyltransferase 3 (LPCAT3) facilitate the esterification and remodeling of PUFAs into membrane phospholipids, rendering them susceptible to oxidative damage (28).

Upon ROS attack, PUFAs are converted into lipid hydroperoxides (PUFA-OOH) (29, 30). If not adequately detoxified, these peroxides disrupt membrane integrity, cause bioenergetic failure, and trigger ferroptotic cell death. In contrast, monounsaturated fatty acids (MUFAs) can attenuate lipid peroxidation by competing with PUFAs for incorporation into membranes, a process involving ACSL3 activity, thus serving as a protective mechanism against ferroptosis (31, 32).

#### 2.1.3 Failure of antioxidant defense systems

The redox homeostasis of the cell is crucial for preventing ferroptosis. Central to this defense is GSH, the most abundant cellular antioxidant, and its associated enzyme GPX4. GPX4 catalyzes the reduction of phospholipid hydroperoxides into non-toxic phospholipid alcohols, preserving membrane integrity (33).

The system Xc^-^ transporter, composed of Solute carrier family 7 member 11(SLC7A11) and Solute carrier family 3, member 2(SLC3A2) subunits, imports cystine in exchange for glutamate, thereby sustaining intracellular cysteine levels essential for GSH synthesis. Disruption of system Xc^-^ activity, whether by extracellular glutamate accumulation or pharmacological inhibition, depletes GSH, reduces GPX4 activity, and sensitizes cells to ferroptosis (34).

Mitochondrial redox balance also plays a critical role, with solute carrier proteins such as Solute carrier family 25 member 11(SLC25A11) and Solute carrier family 25 member 10(SLC25A10) mediating GSH transport across mitochondrial membranes. Loss of mitochondrial antioxidant capacity exacerbates ferroptotic stress (35). Notably, direct inhibition of GPX4, either through genetic deletion or chemical inhibitors, rapidly triggers ferroptosis, highlighting its indispensable role in maintaining neuronal survival under oxidative conditions.

#### 2.1.4 GPX4-independent ferroptosis suppressor pathways

In addition to the canonical GPX4–GSH system, several parallel pathways have been identified that independently suppress ferroptosis. Ferroptosis suppressor protein 1 (FSP1), a flavoprotein localized to the plasma membrane, catalyzes the NAD(P)H-dependent reduction of Coenzyme Q10(CoQ10) to Coenzyme Q H_2_(CoQH_2_), thereby halting lipid peroxidation chain reactions; it can also reduce vitamin K, providing an additional antioxidant defense (36, 37). Structural and pharmacological studies further revealed that FSP1 functions as a dimeric flavoprotein generating 6-hydroxy-FAD with intrinsic anti-ferroptotic activity, while small-molecule inhibitors such as iFSP1 competitively target its NAD(P)H-binding site (38, 39). A second ferroptosis defense pathway involves dihydroorotate dehydrogenase (DHODH), a mitochondrial inner membrane enzyme primarily recognized for its role in pyrimidine biosynthesis. Beyond this metabolic function, DHODH reduces CoQ10 within mitochondria, maintaining redox homeostasis and preventing lipid peroxidation–induced ferroptosis (40). Together, FSP1 and DHODH complement GPX4 to form a multilayered protective network that safeguards cellular and mitochondrial integrity under oxidative stress.

### 2.2 The pathological role of ferroptosis in neurological disorders

Accumulating evidence implicates ferroptosis as a pivotal pathological mechanism across a spectrum of neurological diseases, including stroke, TBI, AD, and PD. The contribution of ferroptosis to neural injury can be attributed to three major interrelated processes: oxidative stress amplification, membrane lipid peroxidation, and iron-dependent neuronal death.

#### 2.2.1 Oxidative stress and iron-driven neurotoxicity

In acute brain injuries such as ischemic and hemorrhagic stroke, as well as TBI, BBB disruption and hemorrhage result in excessive iron deposition in the parenchyma. This iron catalyzes ROS production *via* Fenton chemistry, causing oxidative damage to proteins, nucleic acids, and lipids, and ultimately impairing neuronal function and viability (41).

**Figure 2:**
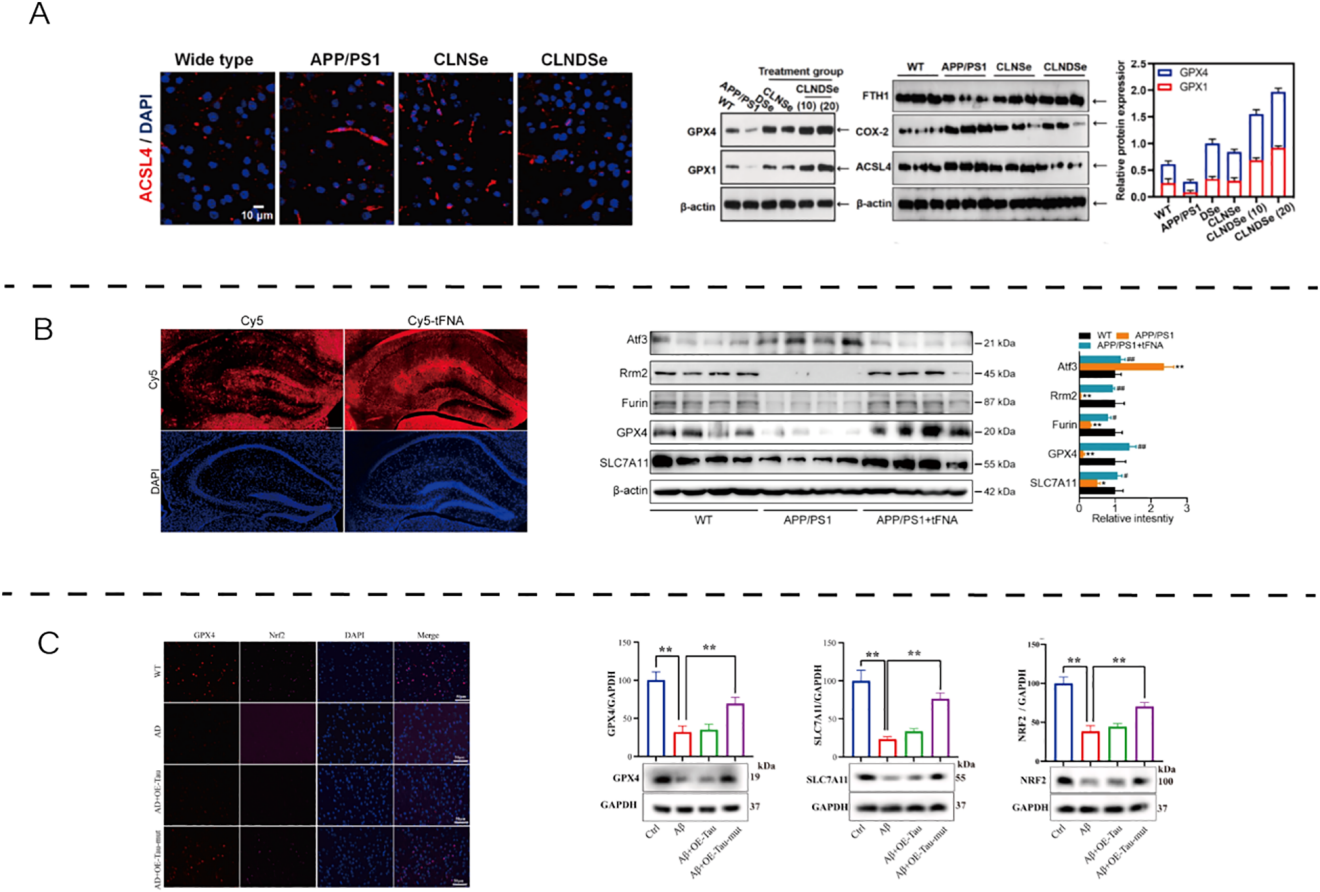
The role and therapeutic modulation of ferroptosis in AD. **(A)** Blood–brain barrier-targeted double selenium nanoparticles restored GPX4 activity, enhanced antioxidant defenses, and improved cognitive outcomes in APP/PS1 mice. Reproduced from Wang et al., 2023, Biomaterials, with permission (42). **(B)** Tetrahedral framework nucleic acids increased cell viability and GSH levels while reducing Fe²^+^, MDA, LDH, and lipid peroxidation in Aβ-treated N2a cells. Reproduced from Tan et al., 2024, Nanobiotechnology, with permission (43). **(C)** Tau K677R mutation alleviated ferroptosis by regulating NCOA4-dependent ferritinophagy and upregulating FTH1 expression, thereby maintaining iron homeostasis and neuronal viability. Reproduced from An et al., 2024, Free Radical Biology and Medicine, with permission (44). APP/PS1, amyloid precursor protein/presenilin-1; WT, wild type; CLNDSe, core–liposome–nanodots selenium nanoparticle; ACSL4, acyl-CoA synthetase long-chain family member 4; GPX4, glutathione peroxidase 4; FTH1, ferritin heavy chain 1; COX2, cyclooxygenase-2; DAPI, 4′,6-diamidino-2-phenylindole; Cy5, cyanine 5; TFNA, tetrahedral framework nucleic acid; NRF2, nuclear factor erythroid 2–related factor 2; FSP1, ferroptosis suppressor protein 1; SLC7A11/xCT, cystine/glutamate antiporter; NCOA4, nuclear receptor coactivator 4; Tau K677R, Tau K677R mutation; β-ACTIN, beta-actin.

In AD models, ferroptosis-related molecular alterations have been demonstrated (**Figure 2**). Representative evidence shows that restoring antioxidant enzymes (e.g., GPX4, xCT, FSP1), modulating ferritinophagy (via NCOA4–FTH1 axis), and improving redox balance collectively mitigate ferroptotic injury and may enhance neuronal viability in AD.

Beyond AD, similar dysregulation of iron homeostasis has been observed in other chronic neurodegenerative diseases such as PD, leading to pathological iron accumulation in vulnerable brain regions (e.g., substantia nigra, hippocampus) (45, 46). A recent review also highlights that oxidative stress, immunological dysfunction, and microbiota shifts collectively shape the pathogenesis of ferroptosis-related neurodegeneration (47). Such elevation of labile iron pools perpetuates oxidative stress, thereby exacerbating synaptic dysfunction and neuronal death.

#### 2.2.2 Lipid peroxidation and membrane disruption

A defining feature of ferroptosis-mediated neural injury is extensive lipid peroxidation. ROS-induced oxidation of PUFA-containing phospholipids compromises membrane fluidity and integrity, resulting in increased membrane permeability, cytoplasmic leakage, and organelle dysfunction. Lipid peroxidation products, such as malondialdehyde (MDA) and 4-hydroxynonenal (4-HNE), further amplify cellular injury by forming cytotoxic adducts with proteins and DNA, thereby propagating neurodegeneration (48).

#### 2.2.3 Neuronal ferroptotic death and functional impairment

Loss of GPX4 activity and GSH depletion render neurons exceptionally vulnerable to ferroptosis. Reduced capacity to detoxify lipid peroxides leads to the activation of ferroptotic death pathways, contributing to neuronal loss and functional deterioration in both acute injuries and chronic neurodegenerative conditions (49).

Preclinical studies have demonstrated that pharmacological inhibition of ferroptosis, using agents such as ferrostatin-1 and liproxstatin-1, or enhancement of antioxidant defenses through N-acetylcysteine supplementation, can significantly reduce infarct volume, improve neurological outcomes, and protect against cognitive decline in various models of stroke, TBI, and neurodegeneration (50).

These findings underscore the critical role of ferroptosis as a unifying mechanism driving neuronal damage and identify it as a promising therapeutic target for the treatment of neurological disorders.

**Figure 3:**
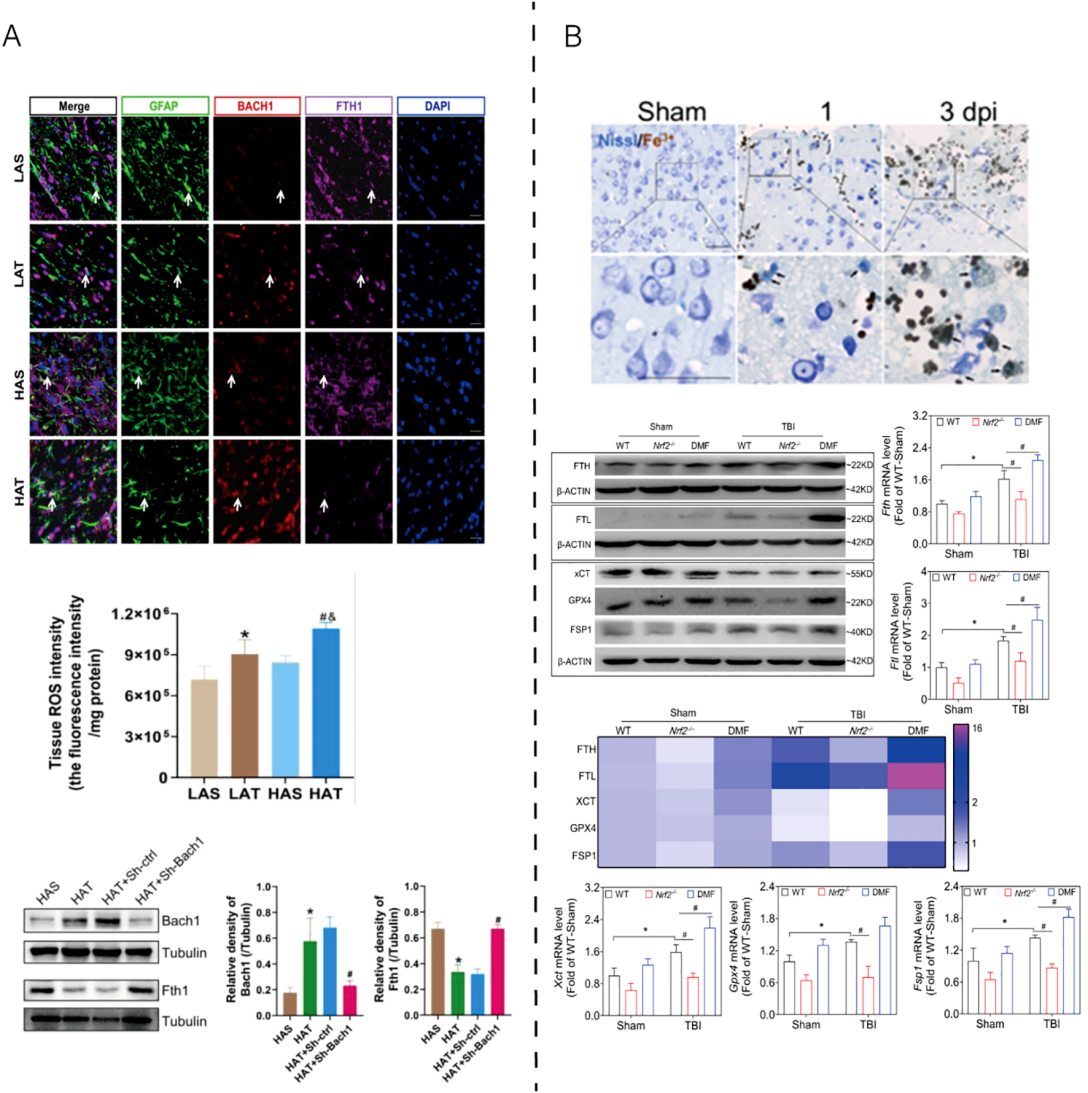
Experimental evidence of ferroptosis regulation in TBI models. **(A)** High-altitude hypoxia aggravates traumatic brain injury by upregulating Bach1, which suppresses antioxidant gene expression and promotes ferroptotic damage. Reproduced from Peng et al., 2025, Cell Death Discov, with permission (51). **(B)** NRF2 activation alleviates TBI-induced ferroptosis by restoring the xCT–GPX4 antioxidant system, enhancing ferritin (FTH/FTL)-mediated iron sequestration, and maintaining redox balance through FSP1–CoQ10-dependent lipid repair. Reproduced from Cheng et al., 2023, Antioxidants, with permission (52). GFAP, glial fibrillary acidic protein; BACH1, BTB and CNC homology 1; FTH1, ferritin heavy chain 1; DAPI, 4′,6-diamidino-2-phenylindole; ROS, reactive oxygen species; β-ACTIN, beta-actin; WT, wild type; Nrf2^-^/^-^, nuclear factor erythroid 2–related factor 2 knockout; DMF, dimethyl fumarate; FTL, ferritin light chain; xCT, cystine/glutamate antiporter (SLC7A11); GPX4, glutathione peroxidase 4; FSP1, ferroptosis suppressor protein 1; Fe²^+^, ferrous iron; Nissl, Nissl staining; dpi, days post injury; LAS, low-altitude sham; LAT, low-altitude TBI; HAS, high-altitude sham; HAT, high-altitude TBI; Tubulin, structural protein used as internal control.

As shown in **Figure 3**, ferroptosis-associated oxidative stress has been implicated in TBI, contributing to secondary neuronal damage. High-altitude hypoxia further aggravates ferroptosis by upregulating Bach1, increasing ROS levels, and reducing Ferritin heavy chain 1 (FTH1) expression, thereby weakening antioxidant defenses. Conversely, NRF2 activation via DMF treatment restores the xCT–GPX4 axis and enhances FSP1–ferritin–mediated iron sequestration, ultimately maintaining redox homeostasis. These findings underscore that ferroptosis in TBI is dynamically regulated by the balance between pro-oxidant (Bach1-driven) and antioxidant (NRF2-dependent) signaling.

### 2.3 Disease-specific differences in ferroptosis across neurological disorders

Ferroptosis manifests with disease-specific features across neurological conditions. In ischemic stroke, cerebral ischemia/reperfusion rapidly triggers iron accumulation, lipid peroxidation, and GPX4/SLC7A11 depression; the ferroptosis inhibitor ferrostatin-1 reduces infarct volume and improves neurobehavioral outcomes in middle cerebral artery occlusion (MCAO) models, consistent with an Protein kinase B/Glycogen Synthase Kinase 3 Beta (AKT/GSK3β)-dependent protection (53). In hemorrhagic contexts, hemin/hemoglobin drives a variant of neuronal ferroptosis with distinct signaling; pharmacologic inhibition and ferroptosis blockers mitigate injury in ICH models, and in subarachnoid hemorrhage, liproxstatin-1 preserves GPX4, downregulates ACSL4/COX-2, and attenuates neurological deficits (54, 55). In traumatic brain injury, ferroptosis contributes to secondary damage; ferrostatin-1 decreases lesion volume and improves long-term sensorimotor/cognitive outcomes (56). In Alzheimer’s disease, neuronal loss of the iron exporter ferroportin precipitates ferroptosis and memory impairment, while liproxstatin-1/ferrostatin-1 rescue Aβ-induced neuronal death and cognitive defects (12). In Parkinson’s disease, dopamine oxidation promotes GPX4 ubiquitination and loss, provoking dopaminergic neuron ferroptosis; restoring GPX4 ameliorates degeneration and motor deficits (57). In multiple sclerosis (MS), patient lesions/Cerebrospinal Fluid (CSF) show iron overload and oxidized phospholipids; late-stage treatment with a ferroptosis inhibitor (UAMC-3203) or delayed anti-ferroptotic therapy in chronic experimental autoimmune encephalomyelitis (EAE) ameliorates disease severity and pathology, underscoring ferroptosis as a targetable driver of progressive MS (58, 59).

These disease-specific patterns suggest that any exosome-based intervention should be disease-tailored to the dominant ferroptosis drivers in each condition, which we consider next.

## 3 Exosome-mediated modulation of ferroptosis in neurological disorders

### 3.1 Stem cell-derived exosomes in modulating ferroptosis in neural cells

Exosomes derived from mesenchymal stem cells (MSCs) and other stem cell populations exhibit notable potential in regulating ferroptotic signaling through delivery of regulatory RNAs and proteins (60) (61). Recent studies have uncovered distinct molecular mechanisms through which these exosomes alleviate oxidative stress, modulate iron homeostasis, and enhance antioxidant defenses in the CNS.

#### 3.1.1 IL-1β-primed MSC-derived exosomes target the HSPA5/GPX4 axis in intracerebral hemorrhage

Li et al. (2024) reported that exosomes derived from MSCs preconditioned with interleukin-1β (IL-1β-Exos) significantly inhibited neuronal ferroptosis in a rat model of intracerebral hemorrhage (ICH). Mechanistically, these exosomes upregulated GPX4, a critical lipid peroxidation-detoxifying enzyme, and heat shock protein A5 (HSPA5), a molecular chaperone that stabilizes GPX4 by preventing its degradation. Additionally, IL-1β-Exos downregulated iron metabolism-related genes, thereby reducing the intracellular labile iron pool and limiting ROS accumulation. Enhanced activity of antioxidant enzymes, including superoxide dismutase (SOD), GSH peroxidase (GSH-Px), and increased GSH levels—further reinforced the suppression of ferroptotic cell death (62).

#### 3.1.2 BMMSC-derived exosomes alleviate SCI *via* IL-17 pathway suppression

Tang et al. (2024) reported that BMMSC-derived exosomes could mitigate ferroptosis and inflammatory injury in spinal cord injury models, partly via modulation of the IL-17 signaling pathway (63). These findings highlight the capacity of BMMSC-Exos to modulate immune–oxidative interplay in ferroptotic cascades and provide insight into their multifaceted neuroprotective mechanisms. Similarly, ADSC-derived exosomes have also been demonstrated to suppress ferroptotic cell death through antioxidant and metabolic reprogramming mechanisms, further emphasizing the therapeutic diversity of stem cell–derived exosomal cargos.

#### 3.1.3 miRNA- and lncRNA-enriched exosomes regulate ferroptosis via multiple signaling axes

In addition to protein regulation, exosomal non-coding RNAs have emerged as powerful post-transcriptional modulators of ferroptosis:

miR-367-3p, delivered via umbilical cord MSC-derived exosomes, targets enhancer of zeste homolog 2 (EZH2), relieving transcriptional repression of SLC7A11, thereby restoring cystine uptake and GPX4 activity (64, 65).

miR-194, from MSC-derived exosomes, suppresses Bach1, activating the Nrf2/HO-1 antioxidant axis, leading to reduced iron-induced oxidative injury (66, 67).

lncGm36569, enriched in exosomes, acts as a ceRNA for miR-5627-5p, upregulating FSP1, a GPX4-independent ferroptosis inhibitor that catalyzes CoQ10-mediated lipid antioxidant activity (68).

miR-19b-3p, carried by adipose-derived stem cell (ADSC) exosomes, targets iron regulatory protein 2 (IRP2), restoring iron balance via upregulation of FPN and downregulation of TfR1, thus reducing ROS and ferroptosis in ICH models (69).

In addition, the tissue origin of MSCs significantly shapes the properties of their exosomes. UCMSC-Exos are obtained non-invasively, with high yield and low immunogenicity, and are enriched in antioxidant miRNAs that enhance GPX4/SLC7A11 signaling (70, 71). BMMSC-Exos, though historically the most studied, require invasive bone marrow aspiration and show donor variability; they carry regulatory miRNAs such as miR-367-3p that suppress ferroptosis through iron metabolism pathways (72). ADSC-Exos are abundant and easily harvested, enriched in metabolic and anti-inflammatory miRNAs, and have been shown to alleviate ferroptosis by activating the NRF2/SLC7A11/GPX4 pathway or modulating the FXR2/ATF3/SLC7A11 axis (73, 74).

As summarized in **Table 1**, stem cell–derived exosomes from multiple sources converge on HSPA5/GPX4, IL-17/GPX4/SLC7A11/ACSL4, and ncRNA-mediated axes to alleviate ferroptosis across CNS injury models.

**Table 1:** Stem cell-derived exosomes in the regulation of ferroptosis in the CNS.

Exosome source	Key cargo	Target molecule/pathway	Neurological model	Biological effects	References
IL-1β-primed MSCs	HSPA5, GPX4	HSPA5/GPX4 axis	Intracerebral Hemorrhage	Enhances GPX4 stability, reduces Fe²^+^ and ROS, increases SOD/GSH, inhibits ferroptosis	(62)
Bone Marrow MSCs	GPX4, xCT, ↓ACSL4	IL-17/GPX4/xCT/ACSL4	SCI	Reduces oxidative stress and inflammation, restores redox homeostasis, suppresses ferroptosis	(63)
Umbilical Cord MSCs	miR-367-3p	EZH2/SLC7A11	Neurodegeneration	Promotes xCT expression, enhances GSH synthesis, inhibits lipid peroxidation	(64)
MSCs	miR-194	Bach1/Nrf2/HO-1	OGD/R injury	Activates antioxidant transcriptional response, protects neurons	(66)
MSCs	lncGm36569	miR-5627-5p/FSP1	Acute Spinal Cord Injury (ASCI)	Enhances CoQ10-mediated detoxification, mitigates ROS and ferroptosis	(68)
ADSCs	miR-19b-3p	IRP2/FPN/TfR1	Intracerebral Hemorrhage	Reduces iron overload, improves antioxidant capacity, prevents ferroptosis-induced neural injury	(69)

MSCs, mesenchymal stem cells; ADSCs, adipose-derived stem cells; ASCI, acute spinal cord injury; SCI, spinal cord injury; OGD/R, oxygen-glucose deprivation/reperfusion; ICH, intracerebral hemorrhage; IL-1β, interleukin-1β; HSPA5, heat shock protein A5; GPX4, glutathione peroxidase 4; FSP1, ferroptosis suppressor protein 1; CoQ10, coenzyme Q10; FPN, ferroportin; TfR1, transferrin receptor 1; FTH1, ferritin heavy chain 1; IRP2, iron regulatory protein 2; ROS, reactive oxygen species; SOD, superoxide dismutase; GSH, glutathione; ACSL4, acyl-CoA synthetase long-chain family member 4; xCT (SLC7A11), cystine/glutamate antiporter; EZH2, enhancer of zeste homolog 2; Bach1, BTB and CNC homology 1; Nrf2, nuclear factor erythroid 2–related factor 2; HO-1, heme oxygenase-1; lncGm36569, long non-coding RNA Gm36569; miR-5627-5p, microRNA-5627-5p.

### 3.2 Comparative roles of exosomes from different cellular origins in ferroptosis regulation

Despite their heterogeneity, exosomes from different cellular sources share common ferroptosis-regulatory features. Most vesicles alleviate oxidative stress by upregulating GPX4/SLC7A11, suppressing lipid peroxidation, and limiting iron overload.

However, source-specific differences are evident. MSC-Exos display broad-spectrum protection, with IL-1β-preconditioned vesicles acting via the HSPA5/GPX4 axis, and engineered ADSC-Exos targeting microglia through the FXR2/ATF3/SLC7A11 pathway (62, 74). Microglia-Exos are strongly phenotype dependent: M2-derived vesicles suppress ferroptosis by delivering miR-124-3p to inhibit NCOA4/ferritinophagy or by activating FUNDC1 mitophagy, whereas M1-Exos may exert opposite effects (75, 76). Neuronal lineage exosomes are less studied, but NSC-Exos carrying CDC42 reduce ACSL4-driven ferroptosis in Parkinson’s models (77). In addition, astrocyte-derived exosomes preserve GPX4 and attenuate hemin-induced ferroptosis (78), while endothelial progenitor exosomes deliver miR-199a-3p to inhibit SP1, thereby suppressing endothelial ferroptosis (79).

Together, these findings indicate that exosomes form a cell-origin–specific yet complementary network against ferroptosis. Representative studies of exosomes from different cellular sources and their ferroptosis-regulatory mechanisms are outlined in **Table 2**.

**Table 2:** Representative studies on exosomes from different cellular origins in ferroptosis regulation.

Exosome source	Key cargo	Target molecule/pathway	Ferroptosis-regulatory effect	Distinctive features	References
MSCs	HSPA5-related proteins; FXR2→ATF3/SLC7A11	HSPA5/GPX4; SLC7A11/GPX4	Inhibit neuronal ferroptosis, reduce lipid peroxidation	Broad protection; enhanced by IL-1β preconditioning; engineered ADSC-Exos target M2 microglia	(62, 74)
Microglia (M2)	miR-124-3p; mitophagy-related cargo	NCOA4/ferritinophagy; FUNDC1 mitophagy	Suppress ferritinophagy, activate mitophagy, attenuate neuronal ferroptosis	Strong phenotype dependency; M2 protective, M1 may aggravate ferroptosis	(75, 76)
Neuronal lineage (NSCs)	CDC42	ACSL4/lipid metabolism	Reduce ACSL4-driven ferroptosis; improve vascular and behavioral deficits in PD	Limited direct evidence; suggest potential role of neuronal lineage exosomes	(77)
Astrocytes	Hypoxia-preconditioned exosomal cargo	GPX4 regulation	Mitigate hemin-induced neuronal ferroptosis	Emerging evidence; first experimental proof of astrocyte-derived exosomes in ferroptosis	(78)
Endothelial progenitors (EPCs)	miR-199a-3p	miR-199a-3p → SP1	Suppress endothelial ferroptosis; reduce lipid peroxidation	Emphasize vascular protection; highlight peripheral–central crosstalk	(79)

MSCs, mesenchymal stem cells; ADSCs, adipose-derived stem cells; NSCs, neural stem cells; M1, M1-polarized macrophage/microglia; M2, M2-polarized macrophage/microglia; ADSC-Exos, adipose-derived stem cell exosomes; IL-1β, interleukin-1β; HSPA5, heat shock protein A5; GPX4, glutathione peroxidase 4; FXR2, fragile X mental retardation syndrome–related protein 2; ATF3, activating transcription factor 3; SLC7A11, solute carrier family 7 member 11; NCOA4, nuclear receptor coactivator 4; FUNDC1, FUN14 domain-containing protein 1; ACSL4, acyl-CoA synthetase long-chain family member 4; SP1, specificity protein 1; miR-124-3p, microRNA-124-3p; miR-199a-3p, microRNA-199a-3p.

## 4 The molecular mechanism underlying exosome-mediated modulation of ferroptosis in neurological injuries

### 4.1 Ferroptosis-related signaling pathways as core targets

Ferroptosis is governed by several critical signaling pathways, including the GPX4–GSH axis, the xCT–SLC7A11 system, the FSP1–CoQ10 pathway, the DHODH–CoQ10 mitochondrial mechanism, and the Nrf2/HO-1 antioxidant response. These interconnected cascades collectively determine neuronal susceptibility to ferroptosis by controlling iron homeostasis, lipid peroxidation, and oxidative defense.

### 4.2 Exosomal miRNAs orchestrating ferroptosis via signaling pathways

Exosomal miRNAs orchestrate ferroptosis regulation by targeting specific nodes in iron metabolism, lipid peroxidation, and antioxidant defense (80). In oxygen-glucose deprivation/reperfusion (OGD/R)-injured hippocampal neurons, exosome-delivered miR-124 from M2 microglia suppresses NCOA4, limiting ferritinophagy and intracellular iron release, thereby reducing ROS and MDA while restoring GSH and cell viability (75). In TBI and ischemia models, miR-124 also downregulates ubiquitin-specific protease 14 (USP14), mitigating injury-related inflammation and proteotoxic stress (81).

miR-367-3p, enriched in human umbilical cord mesenchymal stem cells (hUCMSC) -derived exosomes, inhibits EZH2, relieving transcriptional repression on SLC7A11 (82). This enhances cystine uptake and GSH synthesis, reinforcing the xCT–GSH–GPX4 axis and suppressing ferroptosis. This mechanism is implicated in models of multiple sclerosis and AD.

Additionally, exosomal miR-484 from skeletal muscle stem cells inhibits ACSL4, indirectly enhancing GPX4 activity by limiting PUFA incorporation into phospholipids, thereby attenuating iron-dependent lipid peroxidation (83).

These findings collectively define a modular system wherein distinct exosomal miRNAs modulate upstream and downstream ferroptosis drivers with high specificity and translational potential.

### 4.3 Exosomal proteins and lncRNAs in pathway-specific ferroptosis regulation

Exosomal non-coding RNAs and stress-response proteins enable post-transcriptional and protein-level intervention in ferroptotic signaling. In an ICH model, IL-1β-induced MSC-derived exosomes upregulate HSPA5, which stabilizes GPX4, preventing lipid peroxide accumulation. These exosomes concurrently reduce Fe^2+^, MDA, and ROS, and restore enzymatic antioxidants including SOD and GSH-Px (62).

In SCI, BMMSC-derived exosomes modulate ferroptosis through simultaneous suppression of IL-17 signaling and rebalancing of lipid metabolism. They upregulate GPX4 and SLC7A11, downregulate ACSL4, and attenuate inflammation by decreasing IL-17A, Act1, and IL-17RA expression (63).

The lncGm36569/miR-5627-5p/FSP1 axis, delivered via MSC-derived exosomes, activates a GPX4-independent ferroptosis checkpoint. By derepressing FSP1, it facilitates CoQ10 recycling and membrane repair under oxidative stress, notably in ASCI models (68).

Exosomes from LPS-stimulated M1 microglia reduce GPX4, SLC7A11, and FTH1 in neurons, exacerbating ferroptotic sensitivity (84, 85). Transcriptomic data confirm these M1-derived vesicles drive ferroptosis-linked transcriptional changes, especially in genes controlling iron handling and lipid ROS metabolism (86).

Thus, exosomal cargo from differently primed stem or immune cells can exert either protective or deleterious ferroptotic effects, dependent on the inflammatory or reparative state of the donor cell.

### 4.4 Exosome-mediated antioxidant signaling

Beyond direct targeting of ferroptosis regulators, exosomal cargos activate systemic antioxidant networks that confer neuroprotection (87). In ischemic stroke models, BMSC-derived exosomes enhance Nrf2 nuclear translocation and downstream HO-1, SOD, and catalase expression, restoring redox homeostasis and inhibiting ferroptotic injury (88, 89).

In TBI, hUCMSC exosomes upregulate lncRNA TUBB6, which modulates Nrf2-dependent transcription and suppresses ACSL4, while maintaining GPX4 expression. Mitochondrial morphology and lipid peroxide levels are normalized, indicating structural and biochemical ferroptosis suppression (71).

**Figure 4:**
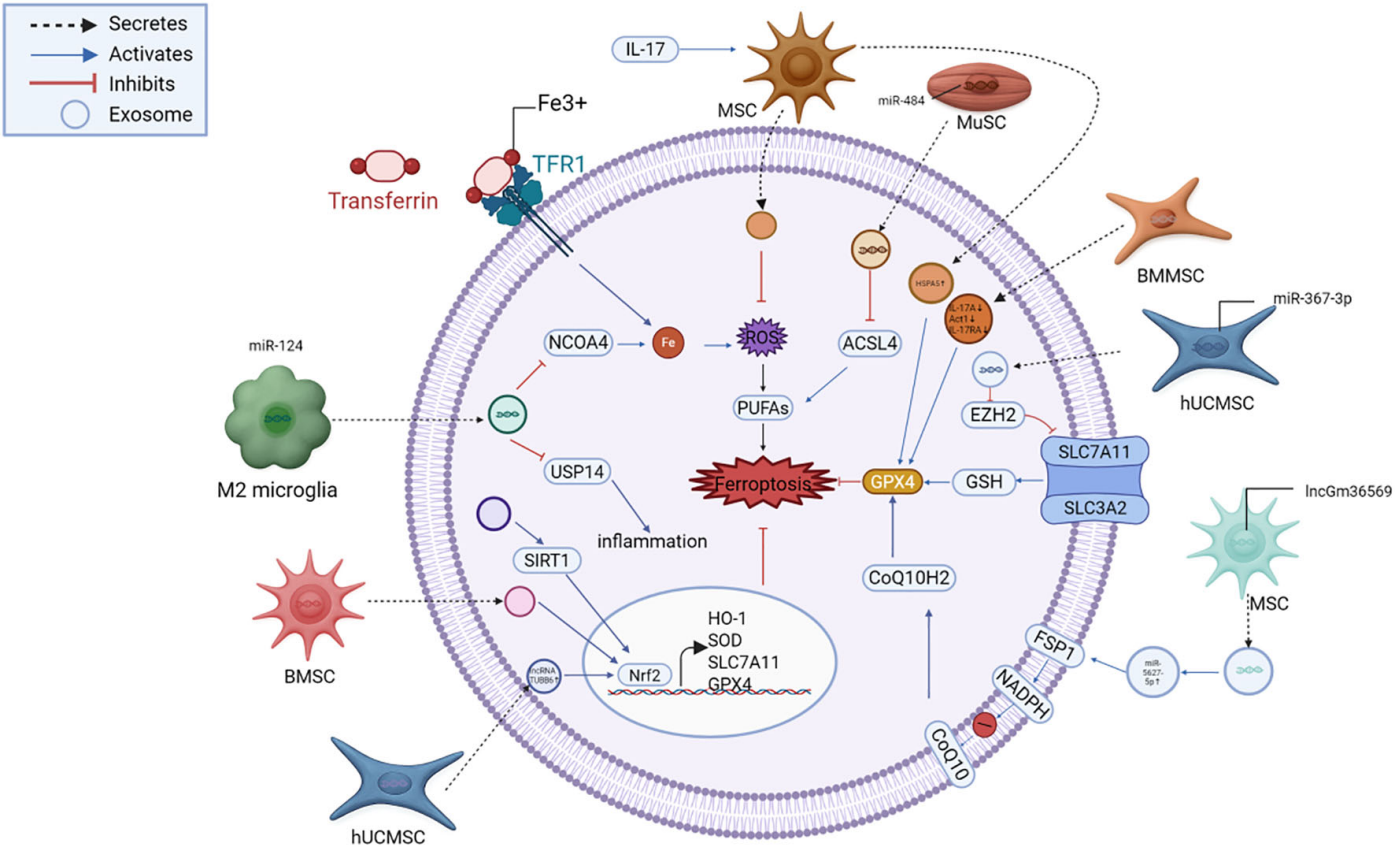
Exosome-mediated suppression of ferroptosis in neural injury. Exosomes deliver functional cargos, including miRNAs (miR-124, miR-367-3p, miR-484), lncRNAs (lncGm36569, TUBB6), and proteins (HSPA5, SIRT1), that modulate key regulators such as NCOA4, EZH2, SLC7A11, GPX4, FSP1, and Nrf2. These pathways converge to suppress iron accumulation, lipid peroxidation, and oxidative stress, thereby protecting neurons from ferroptotic death. MSC, mesenchymal stem cell; ADSC, adipose-derived stem cell; NSC, neural stem cell; ESC, embryonic stem cell; iPSC, induced pluripotent stem cell; microglia, brain-resident immune cell; astrocyte, glial support cell; GPX4, glutathione peroxidase 4; FSP1, ferroptosis suppressor protein 1; DHODH, dihydroorotate dehydrogenase; NRF2, nuclear factor erythroid 2–related factor 2; HO-1, heme oxygenase-1; ROS, reactive oxygen species; Fe²^+^, ferrous iron; GSH, glutathione; SLC7A11 (xCT), cystine/glutamate antiporter; ACSL4, acyl-CoA synthetase long-chain family member 4; COX2, cyclooxygenase-2; TFR1, transferrin receptor 1; NCOA4, nuclear receptor coactivator 4; FTH1, ferritin heavy chain 1.

In aging-related delayed neurocognitive recovery, exosomes boost SIRT1, facilitating Nrf2 nuclear translocation and subsequent HO-1 activation. This reduces free iron, lipid oxidation, and neuronal loss, ultimately improving cognitive outcomes (90). These pathways converge on Nrf2’s master regulatory role in coordinating cellular defense against oxidative ferroptotic damage, with exosomes acting as both inducers and amplifiers of this response. A schematic illustration of these exosome-mediated pathways is presented in **Figure 4**.

## 5 Challenges and application prospects

Exosomes have emerged as promising therapeutic agents for neurological disorders due to their ability to modulate ferroptosis. However, their clinical application faces several challenges that need to be addressed. There exists a diverse array of exosome species, with complex sources. Based on the presence or absence of artificial modifications, exosomes can be categorized into engineered and natural exosomes. Natural exosomes are further classified into those derived from animals and those derived from plants (91). The therapeutic efficacy and safety assessment of exosomes sourced from various origins currently lack systematic analysis. Furthermore, research on the mechanisms underlying exosome function is still insufficient; more in-depth investigations are required regarding cellular uptake, signaling pathways, and targets associated with these vesicles. The prevailing technology for isolating exosomes—ultracentrifugation—can yield them to a certain extent; however, this method often results in low purity levels, requires expensive equipment, and may inadvertently damage the exosomes or lead to their loss (92). Additionally, the relatively limited clinical application of exosome-based therapeutics and inadequate ethical support concerning some human-derived exosomes present significant barriers to translating research outcomes into practical applications.

### 5.1 Biological stability and immune safety

*In vivo*, exosomes are susceptible to rapid clearance by the mononuclear phagocyte system, limiting their therapeutic efficacy (93). Moreover, exosomes may carry immunogenic molecules that trigger immune responses (94).

Seohyun Kim et al. modified exosomes using signal regulatory protein alpha(SIRPα) variants to enhance their ability to evade immune detection and prolong their circulation time. The SIRP-EV achieves active immune escape by mimicking the CD47-SIRPα immune checkpoint signal and significantly extends circulation time through the optimization of surface charge and protein corona control, which reduces non-target retention (95).

A study conducted by the University of Toledo in Toledo, Ohio, USA, demonstrated a dual-mode synergy of “targeting + escape”—where the CD47p110–130 peptide facilitates “escape escort”, and the Arg-Gly-Asp(RGD) peptide provides “targeting guidance”. ExoSmart overcomes the limitations of traditional exosome delivery, presenting a new paradigm for the precise treatment of solid tumors, such as pancreatic cancer (96).

Advanced separation and purification techniques can diminish the presence of immunogenic contaminants, thereby improving the safety and efficacy of exosome-based therapies (97). Furthermore, it was discovered that combining exosomes with biomaterials, such as hydrogels, can facilitate local and sustained release, enhancing their therapeutic effects while minimizing systemic clearance (98–100).

### 5.2 BBB penetration

The BBB presents a significant obstacle for the delivery of therapeutic agents to the CNS. Although exosomes have inherent abilities to cross the BBB, their efficiency remains suboptimal (101). Engineering exosomes with targeting ligands, such as rabies virus glycoprotein peptides that bind to nicotinic acetylcholine receptors, can enhance BBB penetration (102). Exosomes enriched with miRNA have been demonstrated to transiently enhanceBBB permeability by down-regulating tight junction proteins, such as claudin-5 (103). Additionally, external stimuli like focused ultrasound have been employed to transiently disrupt the BBB, facilitating exosome entry (104, 105).

### 5.3 Targeted delivery efficiency

Achieving targeted delivery of exosomes to specific neuronal populations is crucial for maximizing therapeutic outcomes and minimizing off-target effects (106). Surface functionalization of exosomes with antibodies or ligands specific to neuronal markers, such as L1 cell adhesion molecule (L1CAM) or neural cell adhesion molecule(NCAM), can enhance targeting specificity (74, 107). Furthermore, magnetic guidance using superparamagnetic iron oxide nanoparticles incorporated into exosomes allows for spatial control of delivery under an external magnetic field (108). Extracellular vesicles in engineering present a promising option for targeted delivery. A monoclonal antibody that targets the growth-associated protein-43 (GAP43) has been employed to direct extracellular vesicles towards the extracellular environment of damaged neurons in an ischemic stroke model. This approach ensures that the extracellular vesicles accurately deliver their contents to the specific neuronal population intended (109).

### 5.4 Exosome engineering for enhanced therapeutic efficacy

Advancements in exosome engineering have enabled the incorporation of therapeutic molecules, including miRNAs, proteins, and small molecules, to modulate ferroptosis pathways effectively (110). For instance, loading exosomes with miR-124 can downregulate NCOA4, reducing ferritinophagy and iron accumulation. Similarly, exosomes enriched with miR-367-3p can suppress EZH2, leading to upregulation of SLC7A11 and enhanced GSH synthesis. Prof. Li Xukun and his colleagues from Wenzhou Medical University have conducted research on the utilization of exosomes for drug loading and targeted delivery through genetic engineering and chemical modification. They successfully delivered Fibroblast Growth Factor 20(FGF20) for the treatment of ischemic stroke and collaborated with endogenous miRNAs, such as miR-181b-5p, to enhance neural plasticity (111). These modifications can be achieved through electroporation, transfection, or incubation methods (64).

### 5.5 Standardization and scalability of exosome production

For clinical translation, standardized and scalable production of exosomes is essential. Current isolation methods, such as ultracentrifugation and size-exclusion chromatography, have limitations in yield and purity. Emerging techniques like tangential flow filtration and microfluidic-based isolation offer improved scalability and consistency (112, 113). Establishing Good Manufacturing Practice compliant protocols will be critical for regulatory approval and widespread clinical use.

**Figure 5:**
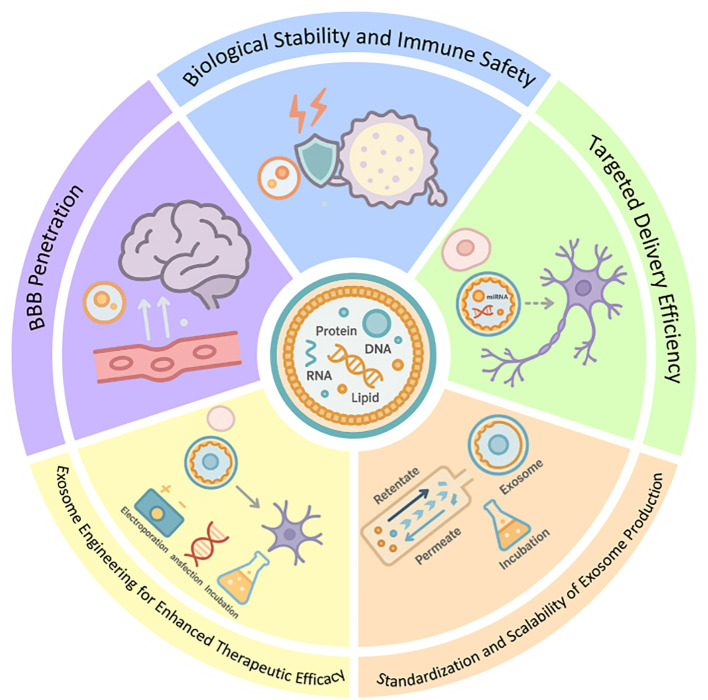
Future improvements and prospects of exosome applications.

A study demonstrates that the combination of Tangential flow filtration (TFF) and Size exclusion chromatography(SEC) can enhance particle concentration by 16.9 times, establishing it as a viable method for mass production (114). Furthermore, researchers from the Department of Pharmacy at Yonsei University in Korea have discovered that hypotonic stimulation and cytochalasin-B therapy can significantly increase exosome yield and drug-carrying capacity (115). Future improvements and translational prospects for exosome-based therapies are depicted in **Figure 5**.

## 6 Safety considerations for exosome-based therapies

### 6.1 Potential safety risks

#### 6.1.1 Off-target effects

Extracellular vesicle surface targeting ligands (such as RVG peptides) may bind to non target cell receptors (such as peripheral nerve nAChR), leading to drug delivery to non target tissues (such as heart, muscle) (102). In addition, the regulatory RNA carried by it (such as miR-181b-5p) may interfere with the normal signaling pathway of receptor cells (such as the PTEN/PI3K-AKT pathway), affecting cell metabolism or proliferation. In acute lymphoblastic leukemia (ALL), miR-181b-5p carried by exosomes is internalized into leukemia cells, upregulated in expression, promoting cell proliferation, migration, and invasion, while inhibiting cell apoptosis (116). Such off target effects may induce organ toxicity, metabolic disorders, or tumor risk.

#### 6.1.2 Immunogenicity concerns

The immune system itself has inherent immunogenicity. Extracellular vesicle membrane proteins may activate host immune responses and trigger a storm of inflammatory factors (117, 118). Residual donor cell DNA/RNA may trigger the TLR signaling pathway, leading to dendritic cell activation and adaptive immune response. In animal models, serum complement activation and neutrophil infiltration can usually be observed after injection of unpurified extracellular vesicles (119).

### 6.2 Strategies to mitigate safety risks​​

#### 6.2.1 Donor cell screening and modification​

To effectively screen donor cells for practical applications, it is essential to select low immunogenicity cell sources, such as autologous MSCs or immune-exempt induced pluripotent stem cells (iPSCs), while avoiding the expression of allogeneic major histocompatibility complex (MHC) molecules. Gene editing techniques, such as CRISPR-Cas9, can be employed to knock out immunogenic genes, exemplified by silencing the B2M gene to eliminate MHC-I expression, thereby reducing immunogenic interference from the source (120–122). Furthermore, engineering modifications, including the display of immune evasion molecules on the cell surface, inhibition of macrophage phagocytosis, introduction of tissue-specific targeting peptides, and enhancement of brain-specific delivery, can also mitigate common risks associated with donor cell utilization (123).

#### 6.2.2 Exosome purification and quality control

During the extraction process of extracellular vesicles, free proteins and apoptotic bodies are typically removed using ultracentrifugation in conjunction with size exclusion chromatography (124, 125). Additionally, anti-CD63/CD81 antibody columns may be employed for purification to ensure the uniformity of exosome subpopulations (126, 127). Furthermore, the quality of extracellular vesicles produced from different batches was monitored through nanoparticle tracking analysis (NTA), Western blotting (WB), and endotoxin level assessments. Together, these procedures establish a standardized quality-control framework that improves batch-to-batch consistency and regulatory readiness.

#### 6.2.3 Off target effect control and loading safety

The quality of extracellular vesicles (EVs) produced from different batches can be significantly enhanced through advanced separation techniques such as size exclusion chromatography (SEC) and density gradient ultracentrifugation. These methods effectively remove unwanted proteins and cytokines that may induce off-target effects. For instance, EV formulations that are depleted of soluble cytokines, such as VEGF-A and Monocyte chemoattractant protein 1 (MCP-1), exhibit enhanced immunomodulatory activity. Such purification techniques ensure that extracellular vesicles retain their therapeutic potential while minimizing adverse reactions to the greatest extent possible (128). Furthermore, electroporation or chemical transfection can be employed to load exogenous cargo onto exosomes. However, these technologies must be meticulously optimized to prevent damage to the EV membrane or alterations in functionality. For example, the CRISPR ribonucleoprotein (RNP) complex was successfully encapsulated into EVs using a protein binding strategy, demonstrating high delivery efficiency (129). Additionally, it is crucial to avoid the direct loading of highly toxic drugs. The quality of the EVs was monitored through NTAandWB, and endotoxin level assessments. Optimized loading conditions and stringent release testing minimize off-target risks while preserving vesicle integrity and therapeutic function, complementing the purification workflow described above.

### 6.3 Clinical translation framework

Before clinical application, drugs typically undergo a comprehensive safety evaluation that primarily assesses their immunotoxicity through the detection of serum complement activity, lymphocyte subsets, and cytokine profiles. Furthermore, organ toxicity is evaluated through histopathological examinations of major organs and long-term monitoring for carcinogenicity. Additionally, administering drugs based on particle count rather than protein dosage ensures consistent quality across batches. These methods are essential for evaluating and validating safety prior to clinical use.

## 7 Conclusions

Ferroptosis has emerged as a key driver of neuronal death in a wide spectrum of neurological disorders, from acute brain injuries to chronic neurodegeneration. In this context, exosomes offer a unique and highly adaptable platform for targeted therapeutic intervention. By leveraging their innate ability to cross the BBB and deliver functional cargo such as regulatory miRNAs, lncRNAs, and proteins, exosomes can modulate core ferroptosis pathways, such as the GPX4-GSH axis, ferritinophagy, and lipid peroxidation, at both transcriptional and post-translational levels. Engineered exosomes further expand this potential through surface ligand modification, cargo enrichment, and responsive delivery systems, enabling precise spatial and molecular targeting within injured neural tissues. Despite this promise, substantial barriers remain, including limited *in vivo* stability, heterogeneity in large-scale production, and the need for validated clinical-grade manufacturing and safety frameworks. Moving forward, the convergence of nanotechnology, molecular neuroscience, and synthetic biology will be essential to transform exosome-based ferroptosis modulation from a preclinical concept into a clinically actionable therapy. With continued interdisciplinary innovation, exosomes are poised to become a next-generation strategy for combating ferroptosis-driven brain injury and advancing the frontier of neuroprotective medicine.

## Glossary

AD: Alzheimer’s disease
PD: Parkinson’s disease
TBI: Traumatic brain injury
ADI: Alzheimer’s Disease International
ROS: Reactive oxygen species
BBB: Blood–brain barrier
CNS: Central nervous system
GPX4: Glutathione peroxidase 4
GSH: Glutathione
Fe²⁺: Ferrous iron
Fe³⁺: Ferric iron
OH: Hydroxyl radicals
FPN: Ferroportin
HIFs: Hypoxia-inducible factors
NTBI: Non-transferrin-bound iron
NCOA4: Nuclear receptor coactivator 4
PUFAs: Polyunsaturated fatty acids
ACSL4: Acyl-CoA synthetase long-chain family member 4
ACSL3: Acyl-CoA synthetase long-chain family member 3
LPCAT3: Lysophosphatidylcholine acyltransferase 3
PUFA-OOH: Lipid hydroperoxides
MUFAs: Monounsaturated fatty acids
SLC7A11/xCT: Solute carrier family 7 member 11
SLC3A2: Solute carrier family 3 member 2
SLC25A11: Solute carrier family 25 member 11
SLC25A10: Solute carrier family 25 member 10
MDA: Malondialdehyde
4-HNE: 4-Hydroxynonenal
MSCs: Mesenchymal stem cells
ICH: Intracerebral hemorrhage
HSPA5: Heat shock protein A5
IL-1β: Interleukin-1β
SOD: Superoxide dismutase
GSH-Px: Glutathione peroxidase
BMMSC-Exos: Exosomes derived from bone marrow MSCs
ADSCs: Adipose-derived stem cells
ASCI: Acute spinal cord injury
FSP1: Ferroptosis suppressor protein 1
hUCMSCs: Human umbilical cord mesenchymal stem cells
EZH2: Enhancer of zeste homolog 2
CoQ10: Coenzyme Q10
CoQH₂: Coenzyme Q H₂
IRP2: Iron regulatory protein 2
FTH1: Ferritin heavy chain 1
BMSCs: Marrow-derived mesenchymal stem cells
Nrf2: Nuclear factor erythroid 2–related factor 2
KEAP1: Kelch-like ECH-associated protein 1
HO-1: Heme oxygenase-1
FGF2: Fibroblast growth factor 2
DHODH: Dihydroorotate dehydrogenase
DMF: Dimethyl fumarate
Bach1: BTB and CNC homology 1
PTGS2: Prostaglandin-endoperoxide synthase 2
miRNA: MicroRNA
lncRNA: Long non-coding RNA
PDENs: Plant-derived exosome-like nanoparticles
VEGF: Vascular endothelial growth factor
VEGF-A: Vascular endothelial growth factor A
PINK1: PTEN-induced kinase 1
MFF: Mitochondrial fission factor
FUNDC1: FUN14 domain containing 1
NADPH: Nicotinamide adenine dinucleotide phosphate
RNS: Reactive nitrogen species
mPTP: Mitochondrial permeability transition pore
DOELNs: Dendrobium officinale exosome-like nanoparticles
EVs: Extracellular vesicles
DOX: Doxorubicin
TNF-α: Tumor necrosis factor alpha
IL-6: Interleukin-6
IL-17: Interleukin-17
IL-17RA: Interleukin-17 receptor A
eIF2α: Eukaryotic translation initiation factor 2α
OGD/R: Oxygen–glucose deprivation/reoxygenation
rTMS: Repetitive transcranial magnetic stimulation
ATF3: Activating transcription factor 3
FXR2: Fragile X mental retardation syndrome-related protein 2
MCP-1: Monocyte chemoattractant protein 1
EVs: Extracellular vesicles
RNP: Ribonucleoprotein
CRISPR: Clustered regularly interspaced short palindromic repeats
NTA: Nanoparticle tracking analysis
WB: Western blot
TFF: Tangential flow filtration
SEC: Size exclusion chromatography
FGF20: Fibroblast growth factor 20
SIRPα: Signal regulatory protein alpha
RGD: Arg-Gly-Asp
L1CAM: L1 cell adhesion molecule
NCAM: Neural cell adhesion molecule
GAP43: Growth-associated protein-43
USP14: Ubiquitin-specific protease 14
MCAO: Middle cerebral artery occlusion
AKT: Protein kinase B
GSK3β: Glycogen synthase kinase 3 beta
MS: Multiple sclerosis
CSF: Cerebrospinal Fluid
EAE: Experimental autoimmune encephalomyelitis
